# Static and dynamic adsorption of a gemini surfactant on a carbonate rock in the presence of low salinity water

**DOI:** 10.1038/s41598-023-38930-z

**Published:** 2023-07-24

**Authors:** Shams Kalam, Sidqi A. Abu-Khamsin, Afeez Olayinka Gbadamosi, Shirish Patil, Muhammad Shahzad Kamal, Syed Muhammad Shakil Hussain, Dhafer Al-Shehri, Emad W. Al-Shalabi, Kishore K. Mohanty

**Affiliations:** 1 Department of Petroleum Engineering, College of Petroleum Engineering & Geosciences, King Fahd University of Petroleum & Minerals, 31261 Dhahran, Saudi Arabia; 2 Centre for Integrative Petroleum Research, College of Petrolcxeum Engineering & Geosciences, King Fahd University of Petroleum & Minerals, 31261 Dhahran, Saudi Arabia; 3 Petroleum Engineering Department, Research and Innovation Center on CO2 and Hydrogen (RICH), Khalifa University, PO BOX 127788, Abu Dhabi, United Arab Emirates; 4 Hildebrand Department of Petroleum and Geosystems Engineering, The University of Texas at Austin, Austin, TX USA

**Keywords:** Chemistry, Materials science

## Abstract

In chemical enhanced oil recovery (cEOR) techniques, surfactants are extensively used for enhancing oil recovery by reducing interfacial tension and/or modifying wettability. However, the effectiveness and economic feasibility of the cEOR process are compromised due to the adsorption of surfactants on rock surfaces. Therefore, surfactant adsorption must be reduced to make the cEOR process efficient and economical. Herein, the synergic application of low salinity water and a cationic gemini surfactant was investigated in a carbonate rock. Firstly, the interfacial tension (IFT) of the oil-brine interface with surfactant at various temperatures was measured. Subsequently, the rock wettability was determined under high-pressure and high-temperature conditions. Finally, the study examined the impact of low salinity water on the adsorption of the cationic gemini surfactant, both statically and dynamically. The results showed that the low salinity water condition does not cause a significant impact on the IFT reduction and wettability alteration as compared to the high salinity water conditions. However, the low salinity water condition reduced the surfactant’s static adsorption on the carbonate core by four folds as compared to seawater. The core flood results showed a significantly lower amount of dynamic adsorption (0.11 mg/g-rock) using low salinity water conditions. Employing such a method aids industrialists and researchers in developing a cost-effective and efficient cEOR process.

## Introduction

The demand for oil and gas continues to soar higher due to the ever-increasing global demand for energy^1,2^. Carbonate reservoirs hold 60% of the global oil reserves^3^. Despite the huge resource potential of these reservoirs, they are characterized by several complexities such as fractures, heterogeneities, and low permeabilities, which limit production^4,5^. Moreover, carbonate reservoirs are typically characterized by high temperature and high salinity conditions. Due to these and other factors, oil recovery from such reservoirs after primary production and waterflooding is low^6^. To recover additional oil, several enhanced oil recovery (EOR) schemes have been developed^7–10^.

Surfactant flooding—a chemical EOR (cEOR) technique has been investigated for its oil recovery efficiency from carbonate rocks^11–16^. Surfactants’ amphiphilic nature makes them capable of modifying the wettability of reservoir rock and lowering oil–water interfacial tension (IFT), which are desirable for oil production^17,18^. Several studies have highlighted the ability of surfactants to improve pore-scale displacement efficiency^19–21^. Nonetheless, surfactants show limited efficiency under harsh reservoir conditions, especially in the case of carbonate reservoirs. High concentrations of divalent ions in the reservoir system cause surfactant precipitation^22–24^. Moreover, partitioning and high retention of surfactants are exacerbated under high salinity conditions, which reduces surfactant efficiency and may render the process uneconomical^25–27^.

Recently, low-salinity waterflooding (LSWF) has been coveted for EOR due to its efficiency^28–31^. The process involves injecting brine of low salinity compared to the formation water. This method is appealing due to its ease of implementation, low cost, and relative simplicity^32^. The major mechanism proposed for oil recovery via the process is the wettability alteration of the reservoir rock system^33^. Other mechanisms postulated include mineral dissolution, formation of the microemulsion, fines migration, pH increase, and multi-ion exchange^34^. However, the application of the process does not significantly influence the fluid–fluid interaction within the rock.

The effectiveness of low salinity water (LSW) and a natural surfactant for EOR in carbonate core plugs was studied^35^. The authors reported IFT reduction (1.02 mN/m) and wettability alteration (31.25°). Besides, the combination of LSW and surfactant resulted in a 12.5% incremental oil recovery over LSWF. Ahmadi et al. evaluated the effects of controlled brine salinity (smart water) on the efficiency of anionic and cationic surfactants for chemical EOR in carbonates^36^. Tests on carbonate cores via spontaneous imbibition at high temperatures indicated that the combination of smart water and 1000 ppm sodium dodecyl sulfate (SDS) recorded 64.5% oil recovery while the combination of smart water with cetyltrimethylammoniumbromide (CTAB) achieved 72% oil recovery. Similarly, Souayeh et al.^37^ noted that the combination of carboxylate surfactant with polyethoxylated nonionic surfactant and LSW yielded 74% of the original oil in place (OOIP) from oil-wet limestone cores. Moradi et al. observed that the synergic application of smart water and surfactant yielded 72% OOIP^38^. Undoubtedly, the potential of LSW and surfactant offers a higher oil recovery than EOR method individually.

More recently, cationic gemini surfactants have been courted and investigated for chemical EOR^39^. This unique type of surfactant is characterized by two head groups linked with a spacer^40^. As compared to conventional surfactants, the presence of the spacer enhances the hydrophobicity and functionality of the surfactant^13,41^. This surfactant demonstrates sterling and fascinating properties such as low critical micelle concentration (CMC), high salt and temperature tolerance, good viscoelastic property, the ability to lower IFT and alter the wettability of rock surface^41^. The low CMC of the surfactant implies a low quantity of the surfactant is required to achieve the desired efficiency. However, surfactant loss may occur due to adsorption and phase trapping during the surfactant flooding process.

Surfactant adsorption poses a significant challenge during surfactant flooding and can have a decisive impact on the success of the enhanced oil recovery (EOR) process. The main mechanism behind the surfactants’ adsorption on the surface of the rocks is the electrostatic attraction between the surfactant molecules and the rock surface, which carries an opposite charge^42,43^. Surfactant adsorption results in the depletion of surfactant molecules from the aqueous solution, leading to a decrease in surfactant concentration. Hence, the economic viability and effectiveness of surfactant flooding depend greatly on the surfactant's adsorption. Therefore, anionic surfactants are considered favorable for sandstone reservoirs because of their lower tendency for adsorption on such rocks. In contrast, cationic surfactants are chosen for carbonate rocks as they exhibit reduced adsorption tendencies on these types of formations. Nevertheless, even with the selection of a specific surfactant based on the rock type, the presence of impurities such as clay and silicate minerals can lead to surfactant adsorption^44,45^. Several studies are talking about surfactant adsorption and its reduction^46–52^. The traditional practice is to use an alkali to reduce the adsorption of anionic surfactants on sandstone rocks^43^. Sodium polyacrylate has also been reported as a sacrificial agent to reduce the adsorption of anionic surfactant on sandstone rocks^53^. Another study shows the use of silica nanoparticles to reduce the adsorption of a natural anionic surfactant on different types of rock surfaces, including sandstone, carbonate, and bentonite^54^. There are some studies as well talking about the adsorption reduction of cationic surfactants on carbonate rocks. Ahmadali et al.^55^ revealed that calcium and magnesium cations have a significant inhibitory effect on the adsorption of cationic surfactants on carbonate rocks. A recent study demonstrated that the addition of methylene blue to the aqueous solution of a cationic surfactant resulted in a decrease in the surfactant adsorption on carbonate rocks^52^. Another approach is to use an acidic medium. Such as the use of CO_2_ to acidify the surfactant solution, increasing the positive charge of the carbonate surface^45^. Consequently, the adsorption of cationic surfactant was decreased because of the electrostatic repulsion^45^. A most recent study found that adding formic acid to the aqueous solution of surfactant significantly reduces surfactant adsorption on carbonate rocks^51^.

Most studies talk about conventional monomeric surfactants and the use of alkalis, nanoparticles, or polymers to reduce surfactant adsorption^43^. Herein, the synergistic combination of LSW and a novel, in-house-synthesized, cationic, gemini surfactant was studied to investigate the surfactant adsorption on carbonate rocks. The surfactant used in this study is an in-house developed dicationic amphiphile and it was designed in a way to address the challenges associated with the chemical EOR. For example, the ethoxy groups were introduced between the lipophobic ammonium head and lipophilic alky tail, and due to these ethoxy groups, the surfactant was found to be soluble in low to high-salinity brine. In addition, the insertion of amide group [–NH–(C(O)–] in the chemical structure displayed several advantages such as low toxicity, low CMC, good biodegradability, and intermolecular hydrogen bonding which leads to the high thermal stability of the molecule. It is well known that the spacer group in gemini surfactant plays a significant role and increasing the length of the spacer group leads to improved interfacial and thermal properties. Therefore, we selected a large spacer group (12 carbon) to obtain better physicochemical properties. Moreover, the developed surfactant is cationic, and the carbonate rocks contain a positive charge and due to charge repulsion, the surfactant adsorption can be minimized. The underlying mechanisms of the EOR method were also examined using this surfactant: 1. The IFT of the oil/brine system and 2. The wettability of the rock-fluid interface. To the best of our knowledge, such comprehensive investigations were never reported before. The research objectives have been accomplished by implementing a systematic approach. Firstly, X-ray diffraction (XRD) and X-ray fluorescence (XRF) techniques were utilized to conduct mineralogical and elemental analyses on rock samples, respectively. After that, IFT and wettability measurements were conducted under different levels of salinity. Static and dynamic adsorption tests were performed under different conditions. Dynamic adsorption tests were carried out using an oil-free core flood setup. For the determination of surfactant concentration, the effluents underwent analysis using high-performance liquid chromatography (HPLC).

## Experimental work

### Materials

#### Brine

Synthetic seawater and formation brine were prepared by dissolving several salts in the deionized water. The low salinity water was made by mixing synthetic seawater with deionized water at a ten-fold dilution. Constituents of the three aqueous phases are shown in Table 1.

**Table 1:** Compositions of the aqueous phases.

Salt	Seawater (g/L)^56^	Low-salinity water (g/L)
NaHCO_3_	0.165	0.0165
Na_2_SO_4_	6.339	0.6339
NaCl	41.172	4.1172
CaCl_2_·2H_2_O	2.387	0.2387
MgCl_2_·6H_2_O	17.644	1.764
Total dissolved solids (TDS)	67.71	6.771

#### Crude oil

The properties of crude oil used in this study are listed in Table 2.

**Table 2:** Characteristics of crude oil^56^.

Property	Saturates	Aromatics	Resins	Asphaltenes	Density, g/cm^3^	Viscosity, cP	API
Value	36.2	50	11	2.8	0.87	12.492	31.07

#### Gemini surfactant

**Figure 1:**
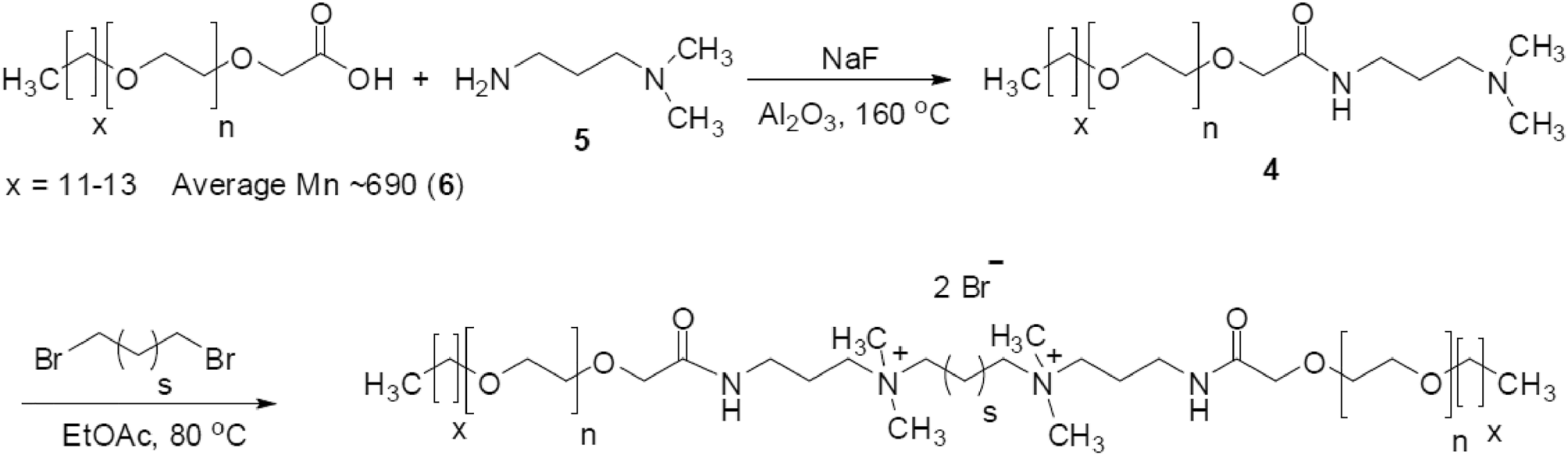
Scheme and structure of the cationic gemini surfactant^57^.

The cationic gemini surfactant employed in this study was synthesized in-house using the route depicted in Fig. 1^57^. The chemical structure was confirmed using FTIR and NMR characterization techniques. The compound is a quaternary ammonium gemini surfactant with bromide as the counterions. Given that the hydrophobic tail and spacer length are both C12, it is a 12-12-12 gemini surfactant. 12-12-12 is also abbreviated as GS12 in this manuscript. The surfactant demonstrates outstanding stability under high salinity and temperature conditions^57^. More details on the synthesis and characterization of the surfactant have been discussed in our previous studies^58,59^.

### Rock characterization

**Figure 2:**
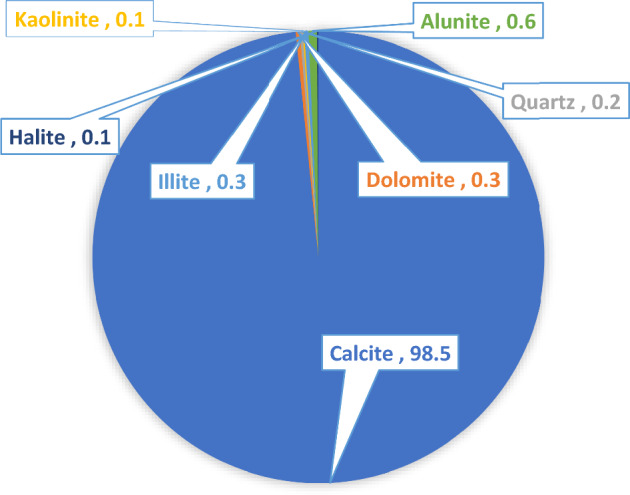
XRD analysis of Indiana limestone core.

Indiana limestone core samples with an average permeability of 10 mD and porosity of 13% were used in this study. Table 3 presents the results obtained from X-ray fluorescence (XRF), while Fig. 2 displays the findings from X-ray diffraction (XRD). XRF shows a higher content of calcium and a lower content of silicon, while XRD depicts a high content of calcite and low content of silicate minerals, indicative of carbonate rocks.

**Table 3:** XRF analysis of Indiana limestone core.

Elements	Percentage, %
Na	0.22
Mg	0.41
Al	0.31
Si	0.44
K	2.99
Ca	94.68
Ti	0.01
Mn	0.05
Fe	0.73
Sr	0.14
Nb	0.01

### IFT measurement

The oil-brine IFT was determined using the pendant drop method in a Kruss Scientific drop shape analyzer. Prior to measurement, bulk fluid was injected into the measuring chamber. The equipment is equipped with a needle (size = 1.57 mm) used to calibrate the measurement. Afterwards, the oil was suspended from the tip of the needle into the bulk fluid. The IFT between the crude oil and LSW, low salinity surfactant solution (LSS), or surfactant in seawater solution (SSW) was measured at three temperatures (25, 50, and 80 °C). The surfactant concentration in LSS and SSW was 1500 ppm.

### Wettability alteration

With Indiana limestone as substrate, the rock-fluid interactions of the various additives were evaluated via contact angle measurement. Each core sample had a diameter of 38 mm and was trimmed to a thickness of 4 mm. Subsequently, the trimmed samples were polished and cleaned with toluene to remove impurities and then dried in an oven at 80 °C. The samples were then aged in the crude oil for 1 week to render the surface oil wet. The cores were treated with LSW as well as LSS and SSW at a surfactant concentration of 1,000 ppm. Afterward, oil with the same properties listed in Table 2 is used as a droplet on the substrate surface, and the contact angles were measured. Each measurement was repeated thrice, and the average value was reported. All experiments were conducted at 80 °C and 1500 psi pressure.

### Static adsorption

To determine the amount of surfactant adsorbed onto a carbonate rock surface, static adsorption tests were performed. After crushing the rock sample into powder, the sample was mixed with the surfactant solution at a ratio of 1:15 and thoroughly shaken for 24 h at ambient conditions. The mixture was subjected to centrifugation at 3000 rpm for 20 min, following which the supernatant was collected and filtered. Subsequently, the unknown concentration of the surfactant was analyzed via high-performance liquid chromatography (HPLC). The sensor used with HPLC was an evaporative light scattering detector (ELSD). Static adsorption was found utilizing Eq. 1.

1 $${\Gamma }_{static}=\left(\frac{{C}_{0}-C}{m}\right).{V}_{s}\times {10}^{-3}$$

where $\Gamma_{static}$ (mg/g-rock) denotes the static adsorption of the surfactant on the rock surface, ${C}_{0}$ (ppm) represents the pre-adsorption surfactant concentration, *C* (ppm) shows the post-adsorption surfactant concentration, ${V}_{s}$ (mL) denotes the surfactant solution volume, and $m$ (g) is the mass of the powdered rock sample.

### Dynamic adsorption

**Figure 3:**
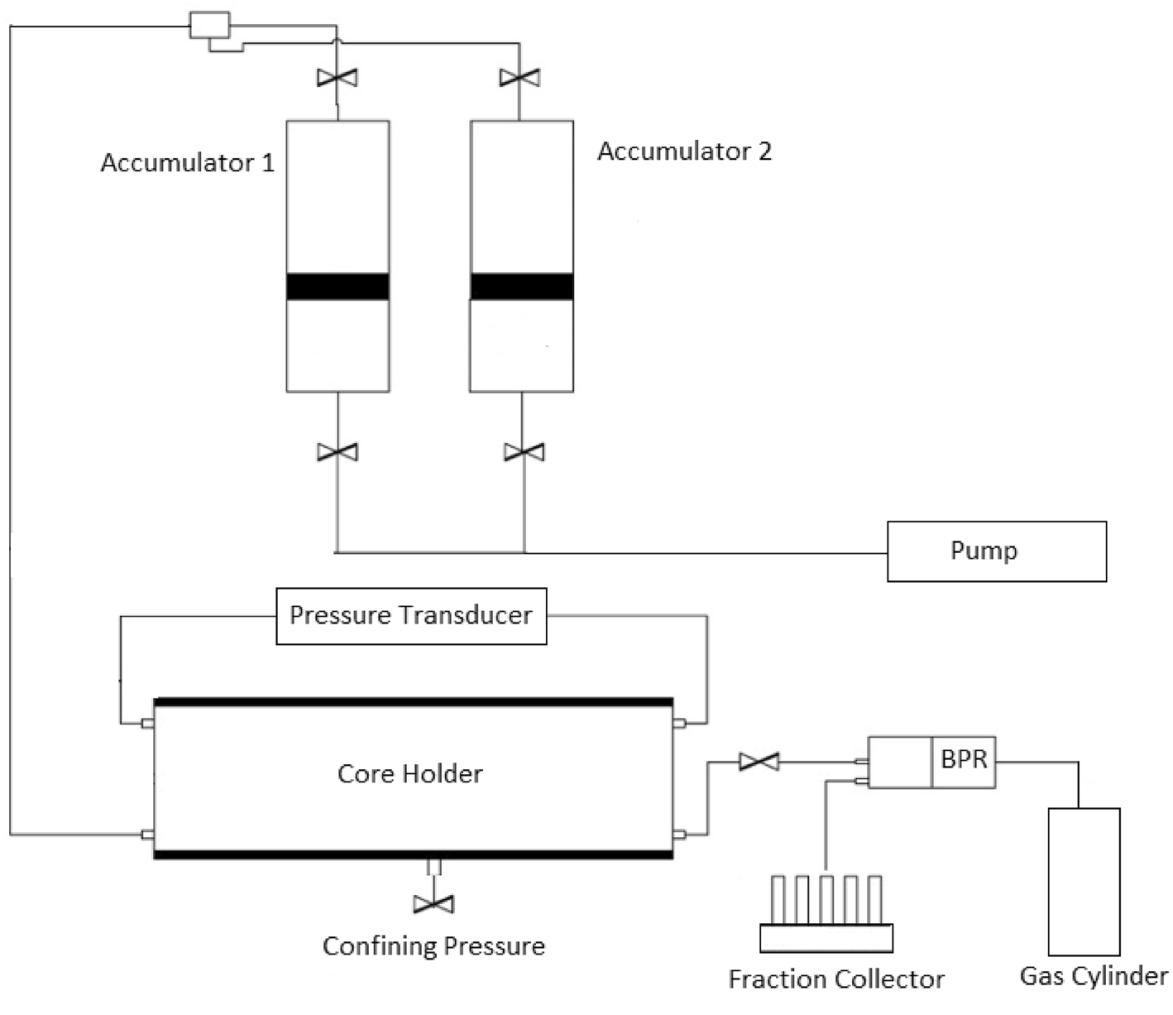
Schematic of dynamic adsorption apparatus.

Two dynamic adsorption tests were performed on Indiana limestone samples in the core-flooding apparatus shown in Fig. 3. Both experiments were conducted under the following conditions: a confining pressure of 2500 psi, a back pressure of 1500 psi, and a liquid injection rate of 0.25 cc/min. The effluent of the core flood was analyzed with HPLC. Dynamic adsorption was calculated using Eq. 2.

2 $$\Gamma_{dynamic} = \frac{{C_{o} V - \sum\limits_{i = 1}^{N} {C_{i} V_{i} } }}{m}$$

where $\Gamma_{dynamic}$ (mg/g-rock) is the surfactant’s dynamic adsorption, C_0_ (ppm) denotes the concentration of the injected surfactant, V (L) is the total volume of injected surfactant solution, Ci (ppm) represents the surfactant concentration, and Vi (L) corresponds to the volume of the effluent collected at different time intervals. The total mass of the dry core sample is denoted as m (g), and the total number of collected effluent samples is represented by N.

## Results and discussion

### IFT results

**Figure 4:**
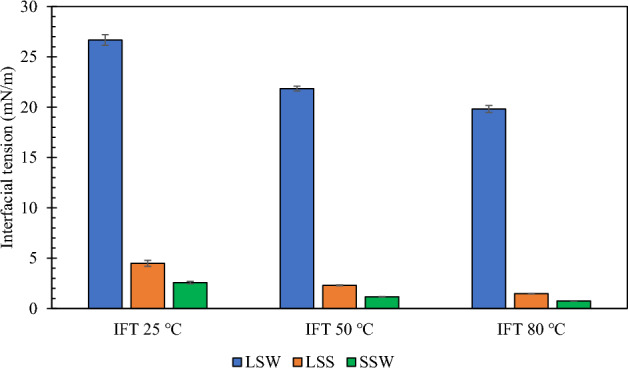
IFT against oil of low-salinity water (LSW), low-salinity surfactant solution (LSS), and surfactant in seawater solution (SSW).

The oil-brine IFT measurements are presented in Fig. 4. With LSW alone, the IFT values were 26.67 $\pm$ 0.53, 21.84 $\pm$ 0.24, and 19.82 $\pm$ 0.35 mN/m at 25, 50, and 80 °C, respectively. This shows the relatively small effect of LSW. This agrees with previous studies that had a similar conclusion^60–62^. On the other hand, the LSS solution caused the IFT to drop drastically at all temperatures reaching 1.48 $\pm$ 0.0355 mN/m at 80 °C. The lowest IFT measured was with SSW, which reached 0.754 $\pm$ 0.015 mN/m at 80 °C. The increased salinity pushes more surfactant molecules to the oil-brine interface leading to lower IFT. As compared to conventional surfactants that precipitate at high salinity conditions, the cationic gemini surfactant utilized in this work is tolerant to high-temperature high-salinity (HTHS) conditions, hence its stability. Additionally, it was observed that the IFT decreases as the temperature condition increases. This can be adduced to the temperature-dependent nature of the formation of rigid interfacial films at the interface. Increasing temperature induces higher mobility at the fluid–fluid interface and increases the entropy of the system. Resultantly, the Gibbs free energy reduces and a lower IFT value is recorded^63^.

### Wettability results

**Figure 5:**
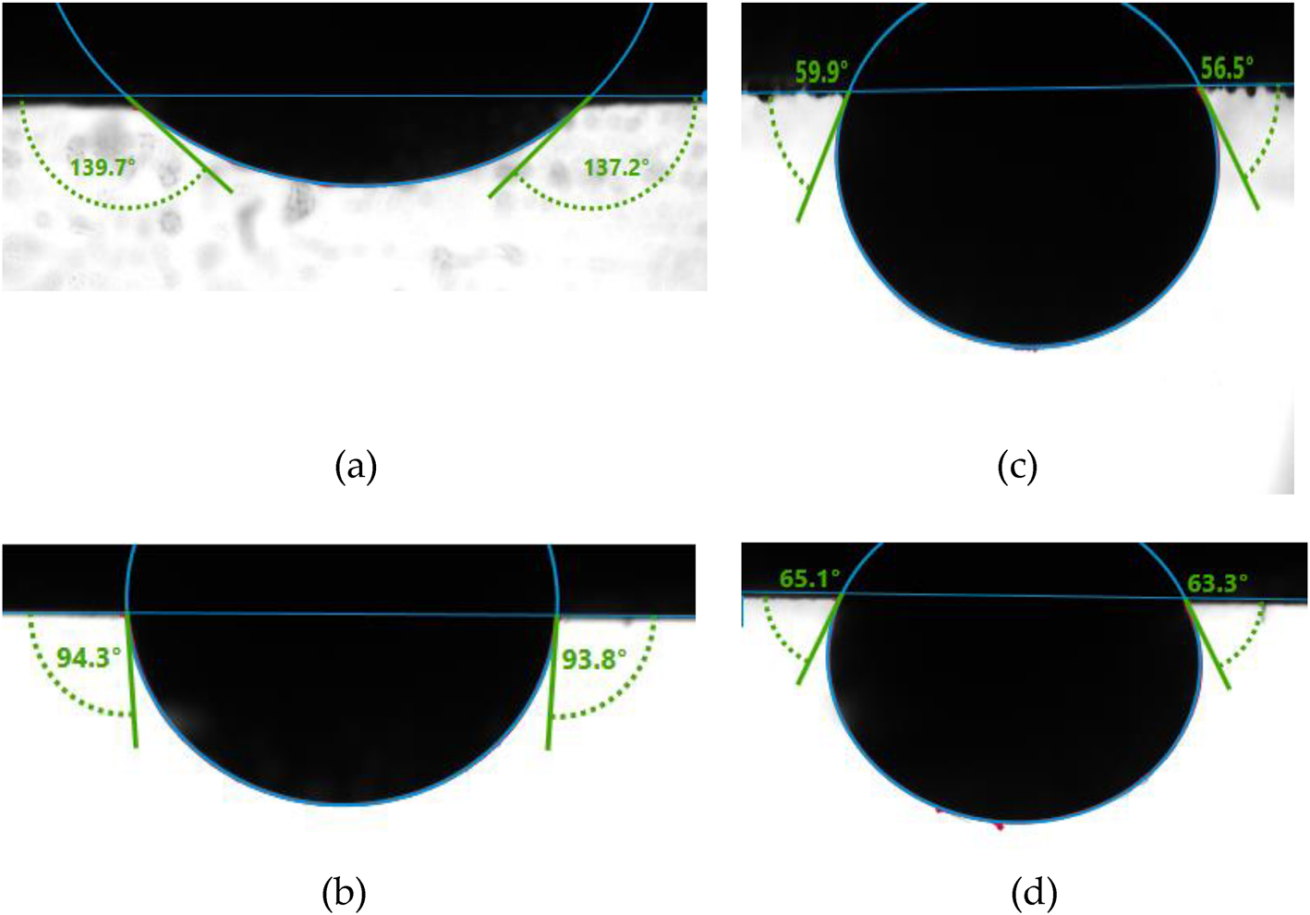
(**a**) The average contact angle between oil and DI water. Wettability alteration of rock samples by (**b**) LSW, (**c**) LSS, and (**d**) SSW solutions.

The wettability of the rock samples aged in crude oil was assessed as depicted in Fig. 5. The average contact angle between oil and DI water (Fig. 5a) was about 138° indicating the oil wetness of the rock. With LSW (Fig. 5b), LSS (Fig. 5c), and SSW (Fig. 5d) the average contact angles were 94°, 60° and 65°, respectively. These results indicate that LSW has a significant impact on rock wettability, shifting it to neutral. The mechanism of carbonate wettability alteration by LSW, especially at high temperatures, has been adduced to multivalent ionic exchange and electrostatic interaction^30,33^. The results also show that the addition of surfactant further altered the rock surface to water wet both under high salinity and low salinity conditions. Wettability alteration results using LSS and SSW were found comparable.

### Adsorption experiments

#### Static adsorption

The static adsorption of the cationic gemini surfactant on the rock is displayed in Table 4, showcasing the results obtained when the surfactant was dissolved in deionized water (DI), high salinity water (HSW), and low salinity water (LSW). A fixed surfactant concentration of 1500 ppm was used in this study because the adsorption plateau was found at around 1000 ppm surfactant concentration^51^. The highest adsorption was found in DI at 1.51 mg/g-rock^50^. The static adsorption was decreased to 0.72 mg/g-rock in HSW and was further reduced to 0.18 in LSW showing a ~ eightfold drop with reference to the base case of DI^50^. In the case of surfactant solution made in DI water, there is no salt present in the aqueous solution. Therefore, there exists a natural interaction between surfactant and the rock surface. The adsorption is high because of the strong electrostatic attraction between the cationic surfactant and the negative binding sites due to silica and clay impurities on the carbonate surface^44,49^. Adsorption of the cationic gemini surfactant was attributed to the formation of an interdigitated bilayer on the carbonate rock^39,40^. On the other hand, in the presence of seawater, there exist monovalent and divalent salts in high concentrations. Therefore, an attraction of divalent ions on the negative binding sites of the carbonate rock surface is expected, leading to a reduction in surfactant adsorption. Ahmadali et al.^55^ also investigated that calcium and magnesium cations have a substantial inhibitory impact on the adsorption of cationic surfactants on carbonates. However, in the case of using low-salinity water conditions, the surfactant adsorption was dramatically reduced. The surfactant adsorption reduction could be due to the expansion of the electric double layer at the solid–liquid interface.

**Table 4:** Static adsorption of cationic gemini surfactant at different conditions.

Condition	Static adsorption (mg/g-rock)
GS12 in DI	1.51
GS12 in HSW	0.72
GS12 in LSW	0.18

Given that the adsorption of GS12 was highest in DI water, the surfactant dissolved in DI water was chosen as the reference for the dynamic adsorption experiment. Further details regarding this experiment will be discussed in the following section.

#### Dynamic adsorption

To study the impact of solution salinity on the dynamic adsorption of GS12 surfactant on Indiana limestone, two different core floods were conducted, one with DI water as solvent as a base case^51^, and the other with LSW. The characteristics of the two flooded core samples are shown in Table 5. In both floods, the core sample was initially fully saturated with the solvent phase, and around 20 PVs of the GS12 solution with a concentration of 1500 ppm was injected at 0.25 cc/min followed by about 5 PVs of the solvent phase.

**Table 5:** Properties of core samples.

Properties	Units	GS12 in DI (base case)^51^	GS12 in LSW
Diameter	mm	38.14	38.15
Length	mm	74.98	74.13
Dry weight	g	182.39	189.88
Bulk volume	ml	85.66	84.74
Grain volume	ml	69.4	71.74
Pore volume	ml	16.26	13
Grain density	g/ml	2.628	2.647
Porosity	%	18.98	15.34
Permeability before flooding	mD	129	155
Permeability after flooding	mD	190	156

**Figure 6:**
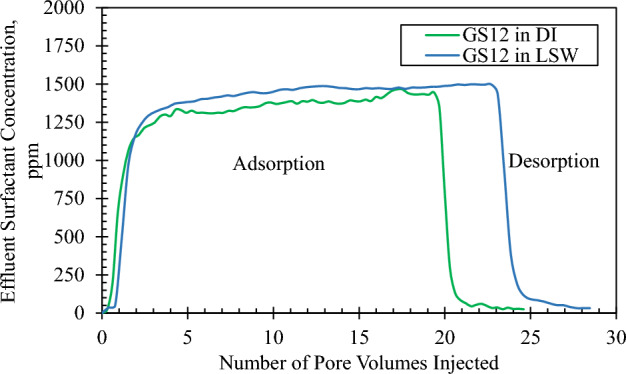
Adsorption–desorption concentration profile of GS12 in deionized water (base case)^51^ and low salinity water.

**Figure 7:**
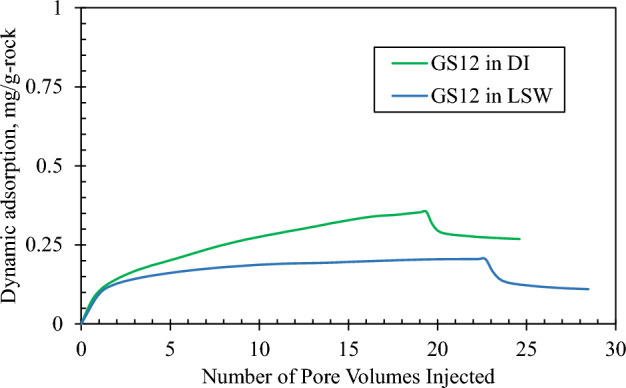
Dynamic adsorption profile of GS12 in deionized water (base case)^51^ and low salinity water.

Figures 6 and 7 show the effluent surfactant concentration and the computed dynamic adsorption profiles for both floods, respectively. The injection of GS12 continued for about 20 pore volumes, followed by low salinity waterflooding to capture the desorption of the surfactant as shown in Fig. 6. Dynamic adsorption of the surfactant made in DI water was 0.269 mg/g-rock on carbonate rock (Indiana Limestone) as shown in Fig. 7^51^. Figure 7 shows that the adsorption of GS12 in LSW was 0.11 mg/g-rock, which is about 60% lower than in DI. In low salinity conditions, the electric double layer at the solid–liquid interface undergoes expansion^64^, which causes a decrease in surfactant adsorption. Nourani et al.^65^ also discovered that the surfactant adsorption was reduced using low salinity conditions. The considerable decrease in surfactant adsorption, amounting to around 60% (from 0.269 to 0.11 mg/g-rock), holds economic significance. One direct consequence is the potential for cost savings through a decreased consumption of gemini surfactant. The cost of manufacturing low-salinity water needs to be determined depending on the geographical location. The utilization of such an approach assists industrialists and researchers in the economical and efficient application of a cEOR process.

## Conclusions

The fluid–fluid and fluid-rock interactions of low salinity water (LSW) with a cationic gemini surfactant (GS12) are summarized as follows.

1. LSW has a small effect on the oil-brine IFT.
2. LSW alters the wettability of oil-wet limestone to intermediate wetting conditions (138° to 94°).
3. The introduction of GS12 surfactant to LSW leads to a substantial decrease in IFT to 1.48 ± 0.0355 mN/m at 80 °C. However, the lowest IFT measured was with SSW, which reached 0.754 ± 0.015 mN/m at 80 °C.
4. LSS and SSW altered the wettability of the oil-wet limestone to the water-wet condition. The corresponding average contact angles were 60° and 65° using LSS and SSW, respectively.
5. With LSS, the static adsorption of the surfactant on the limestone was lowered by about 75% compared with SSW.
6. With LSS, the surfactant’s dynamic adsorption on the limestone was found very low (0.11 mg/g-rock), which illustrates that the use of the gemini surfactant along with LSW will reduce the surfactant adsorption dramatically, therefore, making the chemical EOR process economical and efficient.
7. The synergic combination of LSW and GS12 surfactant shows good potential for recovery of oil in carbonate reservoirs based on good IFT reduction, good wettability alteration, and minimal dynamic adsorption properties.

## Data Availability

The datasets generated during and/or analysed during the current study are available from the corresponding author on reasonable request.

## Abbreviations

cEOR: Chemical enhanced oil recovery
CTAB: Cetyltrimethylammoniumbromide
CMC: Critical micelle concentration
DI: Deionized water
EOR: Enhanced oil recovery
ELSD: Evaporative light scattering detector
GS12: Cationic gemini surfactant with spacer length of C12
HSW: High salinity water
HPLC: High-performance liquid chromatography
HTHS: High-temperature high-salinity
IFT: Interfacial tension
LSWF: Low-salinity waterflooding
LSW: Low salinity water
LSS: Low salinity surfactant solution
OOIP: Original oil in place
PVs: Pore volumes
SSW: Surfactant in seawater solution
TDS: Total dissolved solids
XRD: X-ray diffraction
XRF: X-ray fluorescence

## List of symbols

$\Gamma_{static}$: Surfactant’s static adsorption (mg/g-rock)
$\Gamma_{dynamic}$: Surfactant’s dynamic adsorption (mg/g-rock)
${C}_{0}$: Pre-adsorption surfactant concentration, ppm
*C*: Post-adsorption surfactant concentration (ppm)
*Ci *: Surfactant concentration at time interval i (ppm)
$m$: Mass of the powdered rock sample (g)
*N *: Total number of collected effluent samples
${V}_{s}$: Surfactant solution volume in the static adsorption test (mL)
*V *: The total volume of injected surfactant solution in the dynamic adsorption test (L)
*Vi *: Volume of the effluent collected solution in the dynamic adsorption test at time interval i (L)

## References

[1] El-Masry JF, Bou-Hamdan KF, Abbas AH, Martyushev DAA (2023). Comprehensive review on utilizing nanomaterials in enhanced oil recovery applications. Energies (Basel).

[2] Ogiriki SO, Agunloye MA, Gbadamosi AO, Abdulkashif OO (2018). Exploitation of bitumen from Nigerian tar sand using hot-water/steam stimulation process. Pet. Coal.

[3] Wu J (2022). Improved oil recovery from carbonate reservoirs by tuning injection seawater composition. Energies (Basel).

[4] Dordzie G, Dejam M (2021). Enhanced oil recovery from fractured carbonate reservoirs using nanoparticles with low salinity water and surfactant: A review on experimental and simulation studies. Adv. Colloid Interface Sci..

[5] dos Bastos LS, da Bastos IES, Costa GMN, de Melo SAB (2023). Analyses of interpolant ion effects on smart water core flooding in carbonate. Energies (Basel).

[6] Massarweh O, Abushaikha AS (2023). Application of surfactants in enhancing oil recovery from tight carbonates: Physicochemical properties and core flooding experiments. Geoenergy Sci. Eng..

[7] Guo K, Li H, Yu Z (2016). In-situ heavy and extra-heavy oil recovery: A review. Fuel.

[8] Agi A (2022). Laboratory evaluation to field application of ultrasound: A state-of-the-art review on the effect of ultrasonication on enhanced oil recovery mechanisms. J. Ind. Eng. Chem..

[9] Ngouangna EN (2020). Influence of (3–Aminopropyl) triethoxysilane on silica nanoparticle for enhanced oil recovery. J. Mol. Liq..

[10] Afolabi F, Mahmood SM, Yekeen N, Akbari S, Sharifigaliuk H (2022). Polymeric surfactants for enhanced oil recovery: A review of recent progress. J. Pet. Sci. Eng..

[11] Imuetinyan H, Agi A, Gbadamosi A, Junin R (2022). Extraction, characterization and evaluation of saponin-based natural surfactant for enhanced oil recovery. Arab. J. Geosci..

[12] Imuetinyan H, Agi A, Gbadamosi A, Junin R, Oseh J (2021). Oil-water interfacial tension, wettability alteration and foaming studies of natural surfactant extracted from *Vernonia amygdalina*. Pet. Res..

[13] Kamal MS (2016). A review of gemini surfactants: Potential application in enhanced oil recovery. J. Surfactants Deterg..

[14] Al-Azani K (2022). Oil recovery performance by surfactant flooding: A perspective on multiscale evaluation methods. Energy & Fuels.

[15] Tang W (2023). Synergy of surface modified nanoparticles and surfactant in wettability alteration of calcite at high salinity and temperature. Fuel.

[16] AlZaabi A (2023). Impact of carbonate mineral heterogeneity on wettability alteration potential of surfactants. Fuel.

[17] Bashir A, Sharifi Haddad A, Rafati R (2021). A review of fluid displacement mechanisms in surfactant-based chemical enhanced oil recovery processes: Analyses of key influencing factors. Pet. Sci..

[18] Fogang LT, Kamal MS, Hussain SMS, Kalam S, Patil S (2020). Oil/water interfacial tension in the presence of novel polyoxyethylene cationic gemini surfactants: Impact of spacer length, unsaturation, and aromaticity. Energy Fuels.

[19] Kamal MS, Hussein IA, Sultan AS (2017). Review on surfactant flooding: Phase behavior, retention, IFT, and field applications. Energy Fuels.

[20] Massarweh O, Abushaikha AS (2020). The use of surfactants in enhanced oil recovery: A review of recent advances. Energy Rep..

[21] Chowdhury S, Shrivastava S, Kakati A, Sangwai JS (2022). Comprehensive review on the role of surfactants in the chemical enhanced oil recovery process. Ind. Eng. Chem. Res..

[22] Belhaj AF (2020). The effect of surfactant concentration, salinity, temperature, and pH on surfactant adsorption for chemical enhanced oil recovery: a review. J. Pet. Explor. Prod. Technol..

[23] Abbas AH (2020). Numerical study for continuous surfactant flooding considering adsorption in heterogeneous reservoir. J. King Saud Univ. Eng. Sci..

[24] Gbadamosi A, Radzuan J, Manan M, Agi A, Oseh J (2019). In: Nanotechnology application in chemical enhanced oil recovery: Current opinion and recent advances.

[25] Belhaj, A. F. *et al.* Surfactant partitioning and adsorption in chemical EOR: The neglected phenomenon in porous media. in *SPE-205676-MS, Paper Presented at the SPE/IATMI Asia Pacific Oil & Gas Conference and Exhibition, Virtual, October 2021* 1–35 (2021). 10.2118/205676-MS.

[26] Kalam S, Abu-Khamsin SA, Kamal MS, Patil S (2021). A review on surfactant retention on rocks: mechanisms, measurements, and influencing factors. Fuel.

[27] Kalam S, Abu-Khamsin SA, Kamal MS, Patil S (2021). Surfactant adsorption isotherms: A review. ACS Omega.

[28] Al-Shalabi EW, Sepehrnoori K, Delshad M (2014). Mechanisms behind low salinity water injection in carbonate reservoirs. Fuel.

[29] Katende A, Sagala F (2019). A critical review of low salinity water flooding: Mechanism, laboratory and field application. J. Mol. Liq..

[30] Gbadamosi A (2022). Recent advances on the application of low salinity waterflooding and chemical enhanced oil recovery. Energy Rep..

[31] Kalam S (2021). Data-driven modeling approach to predict the recovery performance of low-salinity waterfloods. Nat. Resour. Res..

[32] Al-Shalabi EW, Sepehrnoori K (2016). A comprehensive review of low salinity/engineered water injections and their applications in sandstone and carbonate rocks. J. Pet. Sci. Eng..

[33] Sagbana PI, Sarkodie K, Nkrumah WA (2022). A critical review of carbonate reservoir wettability modification during low salinity waterflooding. Petroleum.

[34] Al-Bayati A (2022). Wettability alteration during low-salinity water flooding. Energy Fuels.

[35] Dabiri A, Honarvar B (2020). Synergic impacts of two non-ionic natural surfactants and low salinity water on interfacial tension reduction, wettability alteration and oil recovery: Experimental study on oil wet carbonate core samples. Nat. Resour. Res..

[36] Ahmadi S, Hosseini M, Tangestani E, Mousavi SE, Niazi M (2020). Wettability alteration and oil recovery by spontaneous imbibition of smart water and surfactants into carbonates. Pet. Sci..

[37] Souayeh M, Al-Maamari RS, Karimi M, Aoudia M (2021). Wettability alteration and oil recovery by surfactant assisted low salinity water in carbonate rock: The impact of nonionic/anionic surfactants. J. Pet. Sci. Eng..

[38] Moradi S, Isari AA, Bachari Z, Mahmoodi H (2019). Combination of a new natural surfactant and smart water injection for enhanced oil recovery in carbonate rock: Synergic impacts of active ions and natural surfactant concentration. J. Pet. Sci. Eng..

[39] Kalam S (2022). Adsorption mechanisms of a novel cationic gemini surfactant onto different rocks. Energy Fuels.

[40] Khan S (2022). Adsorption study of novel gemini cationic surfactant in carbonate reservoir core—influence of critical parameters. Materials.

[41] Pal N, Hoteit H, Mandal A (2021). Structural aspects, mechanisms and emerging prospects of gemini surfactant-based alternative enhanced oil recovery technology: A review. J. Mol. Liq..

[42] Kalam S, Abu-Khamsin SA, Kamal MS, Patil S (2021). Surfactant adsorption isotherms: A review. ACS Omega.

[43] Kalam S, Abu-Khamsin SA, Kamal MS, Patil S (2021). A review on surfactant retention on rocks: Mechanisms, measurements, and influencing factors. Fuel.

[44] Kalam S (2023). Static adsorption of a novel cationic gemini surfactant: A mineralogical study. Geoenergy Sci. Eng..

[45] Ma K (2013). Adsorption of cationic and anionic surfactants on natural and synthetic carbonate materials. J. Colloid Interface Sci..

[46] Nandwani SK, Chakraborty M, Gupta S (2019). Adsorption of surface active ionic liquids on different rock types under high salinity conditions. Sci. Rep..

[47] Vatanparast H, Shahabi F, Bahramian A, Javadi A, Miller R (2018). The role of electrostatic repulsion on increasing surface activity of anionic surfactants in the presence of hydrophilic silica nanoparticles. Sci. Rep..

[48] Ahmadi MA, Shadizadeh SR (2018). Spotlight on the new natural surfactant flooding in carbonate rock samples in low salinity condition. Sci. Rep..

[49] Kalam S (2022). Adsorption mechanisms of a novel cationic gemini surfactant onto different rocks. Energy Fuels.

[50] Kalam, S. *et al.* Reducing adsorption of a gemini surfactant on carbonate rocks using low salinity water. in *Day 2 Tue, March 14, 2023* (SPE, 2023). 10.2118/214177-MS.

[51] Kalam S (2023). Adsorption reduction of a gemini surfactant on carbonate rocks using formic acid: Static and dynamic conditions. Fuel.

[52] Kalam S (2023). Adsorption characteristics of a cationic gemini surfactant on carbonate outcrops: Adsorption reduction using methylene blue. Energy Fuels.

[53] Koparal, G. B., Sharma, H., Liyanage, P. J., Panthi, K. K. & Mohanty, K. Adsorption of anionic surfactants in sandstones: Impact of sacrificial agents. in *Day 2 Wed, April 21, 2021* (SPE, 2021). 10.2118/200883-MS.

[54] Saxena N, Kumar A, Mandal A (2019). Adsorption analysis of natural anionic surfactant for enhanced oil recovery: The role of mineralogy, salinity, alkalinity and nanoparticles. J. Pet. Sci. Eng..

[55] Ahmadall T, Gonzalez MV, Harwell JH, Scamehorn JF (1993). Reducing surfactant adsorption in carbonate reservoirs. SPE Reserv. Eng..

[56] Gbadamosi A (2023). Evaluating the potential of zwitterionic surfactants for enhanced oil recovery: Effect of headgroups and unsaturation. Energy Fuels.

[57] Hussain SM, Kamal MS, Murtaza M (2019). Synthesis of novel ethoxylated quaternary ammonium gemini surfactants for enhanced oil recovery application. Energies (Basel).

[58] Hussain SMS, Kamal MS, Fogang LT, Patil S (2019). Effect of the number of ethylene oxide units on the properties of synthesized tailor-made cationic gemini surfactants for oilfield applications. J. Mol. Struct..

[59] Hussain SMS, Kamal MS, Murtaza M (2019). Synthesis of novel ethoxylated quaternary ammonium gemini surfactants for enhanced oil recovery application. Energies (Basel).

[60] Alameri, W., Teklu, T. W., Graves, R. M., Kazemi, H. & AlSumaiti, A. M. Wettability alteration during low-salinity waterflooding in carbonate reservoir cores. in *All Days* (SPE, 2014). 10.2118/171529-MS.

[61] Alagic E, Spildo K, Skauge A, Solbakken J (2011). Effect of crude oil ageing on low salinity and low salinity surfactant flooding. J. Pet. Sci. Eng..

[62] Rahevar S (2023). Controlled salinity water flooding and zeta potential: Insight into a novel enhanced oil recovery mechanism. Energy Rep..

[63] Moeini F, Hemmati-Sarapardeh A, Ghazanfari M-H, Masihi M, Ayatollahi S (2014). Toward mechanistic understanding of heavy crude oil/brine interfacial tension: The roles of salinity, temperature and pressure. Fluid Phase Equilib..

[64] Tang G-Q, Morrow NR (1999). Influence of brine composition and fines migration on crude oil/brine/rock interactions and oil recovery. J. Pet. Sci. Eng..

[65] Nourani M, Tichelkamp T, Gaweł B, Øye G (2014). Method for determining the amount of crude oil desorbed from silica and aluminosilica surfaces upon exposure to combined low-salinity water and surfactant solutions. Energy Fuels.

